# Does Sex/Gender Play a Role in Placebo and Nocebo Effects? Conflicting Evidence From Clinical Trials and Experimental Studies

**DOI:** 10.3389/fnins.2019.00160

**Published:** 2019-03-04

**Authors:** Paul Enck, Sibylle Klosterhalfen

**Affiliations:** Department of Internal Medicine VI: Psychosomatic Medicine and Psychotherapy, University Hospital Tübingen, Tübingen, Germany

**Keywords:** sex, gender, placebo, nocebo, randomized controlled trials, experimental studies

## Abstract

Sex has been speculated to be a predictor of the placebo and nocebo effect for many years, but whether this holds true or not has rarely been investigated. We utilized a placebo literature database on various aspects of the genuine placebo/nocebo response. In 2015, we had extracted 75 systematic reviews, meta-analyses, and meta-regressions performed in major medical areas (neurology, psychiatry, internal medicine). These meta-analyses were screened for whether sex/gender differences had been noted to contribute to the placebo/nocebo effect: in only 3 such analyses female sex was associated with a higher placebo effect, indicating poor evidence for a contribution of sex to it in RCTs. This was updated with another set of meta-analyses for the current review, but did not change the overall conclusion. The same holds true for 18 meta-analyses investigating adverse event (nocebo) reporting in RCT in the placebo arm of trials. We also screened our database for papers referring to sex/gender and the placebo effect in experimental studies, and identified 28 papers reporting 29 experiments. Their results can be summarized as follows: (a) Despite higher sensitivity of pain in females, placebo analgesia is easier to elicit in males; (b) It appears that conditioning is effective specifically eliciting nocebo effects; (c) Conditioning works specifically well to elicit placebo and nocebo effects in females and with nausea; (d) Verbal suggestions are not sufficient to induce analgesia in women, but work in men. These results will be discussed with respect to the question why nausea and pain may be prone to be responsive to sex/gender differences, while other symptoms are less. Lastly, we will discuss the apparent discrepancy between RCT with low relevance of sex, and higher relevance of sex in specific experimental settings. We argue that the placebo response is predominantly the result of a conditioning (learning) response in females, while in males it predominantly may be generated via (verbal) manipulating of expectancies. In RCT therefore, the net outcome of the intervention may be the same despite different mechanisms generating the placebo effect between the sexes, while in experimental work when both pathways are separated and explicitly explored, such differences may surface.

## Terminology

The terms sex and gender refer to biological and psychosocial, respectively, origins of differences between women and men. For the purpose of this review these terms will be used interchangeable to describe any difference observed between men and women as it may impact on aspects of experimental medicine and clinical therapeutics, similar to Franconi et al. (2012).

The debate of the terms placebo effect and placebo response have also filled many pages, but will be ignored here for matters of simplicity. Both terms describe the results of a manipulation of treatment by providing an inert drug (in randomized controlled trials, RCT) or manipulating an experimental intervention, either for a whole group or for an individual. It has to be kept in mind, however, that some of these results of the RCT/experiment may be due to factors others than the placebo effect, specifically response biases, the Hawthorn effect, regression to the mean, spontaneous variation of symptoms, and other influences, that need to be controlled for, if at all possible (Enck et al., 2013; Schedlowski et al., 2015). The same ignorance is applied to the differential use of terms nocebo effects and nocebo response, for which the same limitations are valid (Bingel and Placebo Competence Team, 2014).

## The Short History of Placebo Research

Historically, the term “placebo” referred only to the use of inert, pill-like medicines for control of unspecific (not drug-associated) effects in RCT (Kaptchuk, 1998), and for the – occasional – use of similar remedies in everyday-medicine (Fassler et al., 2010). It was later extended to include other and specifically non-medicinal therapies into the arena of evidence-based medicines. Placebo-controlled trials in surgery and other “instrumental” and manual therapies (acupuncture, stimulation techniques such as TENS, TMS, physical therapies, and alike) (Enck, 2018) often use the term “sham” instead, to denote that the provision of placebos in not “inert” any longer: sham surgery for instance can be associated with significant violating of the body’s integrity. The application of the concept of placebos for psychotherapy and therapies alike has received very little and rather late attention and raises substantial controversy nowadays (Blease, 2018) over the question whether psychotherapy is to a large extent only placebo therapy (Gaab et al., 2016), or whether the placebo concept should not be applied at all to psychotherapy (Kirsch, 2005).

The term “nocebo” has a much younger tradition. It was initially describing side effects (adverse events, AE) reported in RCT in the placebo arm of studies, where these AE can only occur as the consequence of mis-attributing symptoms toward the ingested (pill) placebo, or as the consequence of having read and signed AE patient information (Bingel and Placebo Competence Team, 2014). Nocebo effects follow very much the rules for placebo effects both in clinical studies and in experimental settings, as we will describe below, but we will not discuss in more detail the psychobiological and neurophysiological mechanisms behind placebo and nocebo effects – these have been extensively reported by us and others in many reviews in recent years (see for instance Enck et al., 2008, 2013; Elsenbruch and Enck, 2015; Schedlowski et al., 2015).

According to Franconi et al. (2012), female patients are traditionally underrepresented in clinical studies, for different reasons not to elucidate here (e.g., Pinnow et al., 2009). On the other hand specifically in the area on pain, sex differences are well established, both for clinical and for experimental setting (Paller et al., 2009), but also have been found to be variable with sexual orientation and identity (Vigil et al., 2014). In the following sections we will review advances in research over the last decade, with respect to pain and placebo analgesia.

## Sex-Effects on the Placebo Effect in RCT

While age has been shown to consistently affect placebo response rates in a number of clinical conditions investigated during RCT, sex of the patients has rarely been reported to contribute to it. Before 2010, there are only a few papers reporting stronger placebo analgesic responses in male patients (Berkley et al., 2006; Fillingim et al., 2009). Others failed to find sex difference in placebo analgesia, e.g., with tooth extraction (Averbuch and Katzper, 2001), transcutaneous electrical nerve stimulation (Robinson et al., 1998), and an experimental pain test (Olofsen et al., 2005) In a benzodiazepine withdrawal study, female patients had higher placebo responses than males (Saxon et al., 2001). However, sex differences in individual studies (e.g., Kelley et al., 2009) for the irritable bowel syndrome), clinical or experimental, cannot provide sufficient evidence for or reject the assumption of sex differences existing.

In a 2013 systematic review (Weimer et al., 2015) of meta-analyses and systematic review of RCT across most medical subspecialties, based on our JIPS literature database (Enck et al., 2018), we identified only three out of 75 meta-analyses that reported higher placebo response rates in female patients than in males, and in neurological and psychiatric diseases only, namely restless leg syndrome (Ondo et al., 2013), bipolar mania (Yildiz et al., 2011), and schizophrenia (Mallinckrodt et al., 2010). This however, remained not without contradiction by other meta-analyses of the same clinical conditions (Woods et al., 2005; Fulda and Wetter, 2008; Chen et al., 2010), and with analyses of similar size (see Table 1).

**Table 1:** Placebo effects in meta-analyses of randomized controlled trials in selected psychiatric and neurological diseases, with respect to sex influences.

Reference	Disease	No S	Response	Sex
Ondo et al., 2013	RLS	32	Pla	F > M
Mallinckrodt et al., 2010	Schizophrenia	27	Pla	F > M
Yildiz et al., 2011	Bipolar mania	38	Pla	F > M
Woods et al., 2005	Schizophrenia	32	Pla	F = M
Chen et al., 2010	Schizophrenia	31	Pla	F = M
Potkin et al., 2011	Schizophrenia	3ˆ*	Pla	F = M
Agid et al., 2013	Psychosis	50	Pla	F = M
King et al., 2013	Autism, children	1ˆ*	Pla	F = M
Cohen et al., 2010	OCD, anxiety	40	Pla	F = M
Newcorn et al., 2009	ADHD children	10	Pla	F = M
Waxmonsky et al., 2011	ADHD	2ˆ*	Pla	F = M
Buitelaar et al., 2012	ADHD adults	2ˆ*	Pla	F = M
Blom et al., 2014	BED	10ˆ*	Pla	F = M
Brown et al., 1992	Depression	1ˆ*	Pla	F = M
Evans et al., 2004	Depression	4	Pla	F = M
Stein et al., 2006	GAD, MDD	12	Pla	F = M
Bridge et al., 2009	Depression, children	12	Pla	F = M
Brunoni et al., 2009	Depression	41	Pla	F = M
Hunter et al., 2010	Depression	1ˆ*	Pla	F = M

*In further 8 meta-analyses with 287 studies with various gastrointestinal disorders, no gender differences was found, and neither in 3 other meta-analyses 64 studies in different with medical conditions (from Weimer et al., 2015; for references not in our listing, we refer to this paper). No S, Number of RCT included; Response, Placebo (Pla), nocebo (Noc); Sex, M = Males, F = Females; RSL, Rest leg syndrome; MDD, Major Depression Disorder; OCD, Obsessive compulsory disease; ADHD, Attention-deficit hyperactivity disorder; BED, Binge eating disorder; GAD, General anxiety disorder. ^∗^ indicates availability of individualized data.*

This is surprising, given that these 75 analyses – with more than 1,500 RCT included, in more than 40 different diseases and with more than 150,000 patients – covered neurological diseases (Parkinson’s disease, restless leg syndrome, epilepsy), pain syndromes (migraine, neuropathic pain, fibromyalgia), psychiatric diseases (depression, schizophrenia, mania, psychosis, attention-deficit hyperactivity disorder, addiction), gastrointestinal disorders (visceral pain syndromes, inflammatory bowel diseases), and other disorders (asthma, overactive bladder, hypertension, allergy, chronic fatigue, sleep problems).

Adding a few more meta-analyses and conditions published since 2015 (Vase et al., 2015; Ciccozzi et al., 2016; Chen et al., 2017; Imanaka et al., 2017; Razza et al., 2018; Yeung et al., 2018) did not reveal additional evidence for higher placebo response in either sex in any of the diseases. In consequence of this rather clear result, we are forced to conclude that in RCT in the direction and size of the placebo response is not related to the sex of the patients (Weimer et al., 2015).

## Sex Effects on the Nocebo Response in RCT

The number of all papers including the term “nocebo” in our database is 431, of which only 12 (2.7%) refer to sex or gender – implying that in the discussion of the nocebo effects much less attention is paid to sex differences. The database contains 18 meta-analyses on nocebo effects in RCT, covering more than 500 RCT with more than 25,000 patients, but excluding meta-analyses with children and adolescents, papers comparing two or more treatment modes for one condition, or with one treatment mode for more than one disease, and all experimental studies. All of these are in relation to neurological and psychiatric disorders (see Table 2).

**Table 2:** Nocebo effects (adverse events) in meta-analyses of randomized controlled trials, with respect to sex influences.

Reference	Disease	No S	Response	Sex
Meister et al., 2017	Depression	23	Noc	F > M
Zis and Mitsikostas, 2015	Alzheimer	20	Noc	F > M
Dodd et al., 2018	Bipolar disorder	9	Noc	F = M
Hauser et al., 2012	Fibromyalgia	18	Noc	F = M
Mitsikostas et al., 2012	Fibromyalgia	16	Noc	F = M
Dodd et al., 2015	Major depression	20	Noc	F = M
Papadopoulos and Mitsikostas, 2010	MS: DMT	55	Noc	F = M
Papadopoulos and Mitsikostas, 2010	MS: ST	44	Noc	F = M
Papadopoulos and Mitsikostas, 2012	Neuropathic pain	12	Noc	M > F

*No S, Number of RCT included; Response, Placebo (Pla), nocebo (Noc); Sex, M = Males, F = Females; MDD, Major Depression Disorder; CIDP, Chronic inflammatory demyelinating polyneuropathy; MS, Multiple Sclerosis; ST, Symptomatic treatment; DMT, Disease modifying treatment.*

As with the placebo effect, in only three papers an association of sex and the report AE and study termination due to AE was noted: in two meta-analysis the nocebo effect was higher in women (Zis and Mitsikostas, 2015; Meister et al., 2017), in one it was higher in men (Papadopoulos and Mitsikostas, 2012). This leaves us with a similar conclusion as above, that in RCT the direction and size of the nocebo response may not be related to the sex of the patients. It neither seems to be related to age of the patients, as two analyses showed higher AE reports in younger patients (Mitsikostas et al., 2012; Dodd et al., 2018), whereas another two noted higher responses in the elderly (Papadopoulos and Mitsikostas, 2010; Zis and Mitsikostas, 2015). Unfortunately however, most studies neither reported sex nor age as determining factors of the nocebo effect in RCT, either because it was not possible due to small numbers for meta-regressions, or it was not of interest to the authors.

## Sex Differences in Experimental Placebo and Nocebo Studies

The situation is entirely different when placebo experiments are planned to evaluate the mechanisms behind the placebo/nocebo effects seen in RCT. Here recruitment of patients or volunteers can be planned based on a balance sex distribution, and eventually even matched for other social and biological variables, e.g., age, BMI, status etc., depending on the underlying hypotheses. Unfortunately, sex-balanced studies have one disadvantage that is often either ignored or has led to dismissal of female test persons altogether, that is the need for assessment and adjustment of female participants according to their menstrual cycle, e.g., with pain studies (Iacovides et al., 2015). In animal work, not only in placebo research, this has vastly abandoned including female animals at all in many studies (Couzin-Frankel, 2014). Surprisingly, even in experiments with patients the sex of patients is sometimes not reported (e.g., Petersen et al., 2012).

A recent systematic review (Vambheim and Flaten, 2017) identified 18 experiments in 17 papers (among more than 500 experiments, according to our JIPS database) reported in healthy volunteers in which sex as a contributing factor was either investigated purposely, or occurred incidentally with the data evaluation. To these 18 experiments we added 9 further experiments in healthy volunteers and 2 in different patient groups (Liccardi et al., 2004; Skyt et al., 2018).

## Experimental Placebo Studies

A total of 18 experiments were performed on placebo responses in healthy volunteers (Table 3), nearly an equal part showed either stronger responses in males (*N* = 7) and in females (*N* = 6), and 5 showed no sex differences, leaving the question unanswered. Of the three experimental studies in patients, two showed stronger placebo responses in females while one was inconclusive.

**Table 3:** Placebo experiments reporting sex in healthy volunteers either by verbal induction or conditioning of the response (data in part from Vambheim and Flaten, 2017, supplemented by further studies).

				Inter-
Reference	No (Fem)	Method	Condition	vention	Sex
Aslaksen and Flaten, 2008	63 (22)	Verbal	Pain	Pla	M > F
Flaten et al., 2006	84 (47)	Verbal	Pain	Pla	M > F
Aslaksen et al., 2011	33 (17)	Verbal	Pain	Pla	M > F
Bjorkedal and Flaten, 2011	23 (7)	Verbal	Pain	Pla	M > F
Butcher and Carmody, 2012	20 (10)	Verbal	Pain	Pla	M > F
Abrams and Kushner, 2004	41 (25)	Verbal	Distress	Pla	M > F
Oken, 2008	40 (20)	Verbal	Cognition	Pla	M > F
Kotsis et al., 2012	36 (18)	Verbal	Visceral pain	Pla	F = M
Roderigo et al., 2017	60 (30)	Verbal	Visceral pain	Pla	F = M
Theysohn et al., 2014	30 (15)	Verbal	Visceral pain	Pla	F = M
Weimer et al., 2012	64 (32)	Verbal	Nausea	Pla	F = M
Horing et al., 2013	32 (16)	Conditioning	Nausea	Pla	F = M
Krummenacher et al., 2014	49 (23)	Verbal	Pain	Pla	F > M
Colloca et al., 2015	109 (54)	Verbal	Pain	Pla	F > M
Haltia et al., 2008	24 (12)	Verbal	Dop response	Pla	F > M
Klosterhalfen et al., 2005	24 (12)	Conditioning	Nausea	Pla	F > M
Stockhorst et al., 2014	24 (12)	Conditioning	Nausea	Pla	F > M
Weimer et al., 2013	64 (32)	Verbal	Rot.tolerance	Pla	F > M

*No (Fem), Total number of volunteers included (number of females); Intervention, Placebo (Pla), nocebo (Noc); Sex, M = males, F = Females.*

It should be noted, however, that 12 of the 18 studies on this group are from three laboratories only: 4 from the Flaten lab in Tromsö, Norway, 3 from the Elsenbruch lab in Essen, Germany and 5 from our Düsseldorf/Tübingen labs, the remaining six are from six different labs around the world, indicating that except in these three labs, sex effects were probably accidental finding but not the focus of research. In the three laboratories providing more than one study, one group showed a male predominance, one a female predominance, and one found consistently no sex differences. It seems from the distribution in Table 3 that there is a trend for placebo analgesia to be more effective in males, while with experimentally induced nausea females report higher placebo responses. Whether this is due to a laboratory-specific bias or specific to the clinical condition (pain or visceral pain versus nausea, for instance), or to different methods of placebo induction (verbal instruction versus conditioning), cannot be answered due to the small number of studies.

It is noteworthy though that placebo conditioning experiments have never worked for visceral pain (Sigrid Elsenbruch, personal communication); none is reported in the literature so far, despite own and other’s attempts. Taking visceral pain out of the equation, it appears that verbal induction of analgesia works better in men than in women.

Important to note also is the fact that the Colloca et al. (2015) study used oxytocin for support the placebo effect, which is known to work specifically well in females, and may explain the paradoxical finding – compared to all other placebo analgesia studies that reported higher responses in males. The Krummenacher et al. (2014) study was performed in children, so that data are not easily transferable to adults (Weimer et al., 2013).

## Experimental Nocebo Studies

Table 4 lists the 8 experiments performed to induce a nocebo reaction in healthy volunteers; here the distribution seems cleared: Five of the eight studies, and in addition the only patient study reports higher nocebo responses in females than in males, and only 1 male predominance; two remain inconclusive.

**Table 4:** Nocebo experiments reporting sex in healthy volunteers either by verbal induction or conditioning of the response (data in part from Vambheim and Flaten, 2017, supplemented by further studies).

Reference	No (Fem)	Method	Condition	Condition	Sex
Swider and Babel, 2013	84 (42)	Conditioning	Pain	Noc	F > M
Aslaksen et al., 2015	111 (76)	Verbal	Pain	Noc	F > M
Klosterhalfen et al., 2009 (1)	48 (24)	Conditioning	Nausea	Noc	F > M
Faasse et al., 2015	82 (51)	Conditioning	AE	Noc	F > M
Lorber et al., 2007	86 (51)	Conditioning	AE	Noc	F > M
Elsenbruch et al., 2019	60 (30)	Verbal	Visceral pain	Noc	F = M
Stumpf et al., 2016	100 (50)	Verbal	Itch	Noc	F = M
Klosterhalfen et al., 2009 (2)	48 (24)	Verbal	Nausea	Noc	M > F

*No (Fem), Total number of volunteers included (number of females); Intervention, Placebo (Pla), nocebo (Noc); Sex, M = males, F = Females; AE, Adverse events.*

It is noteworthy that among the six with stronger responses in females, four are conditioning studies, as are two of the placebo studies (see Table 3). This underlines our assumption that conditioning may work specifically well in females. When we combine the experimental placebo and the nocebo studies in healthy volunteers and compute a chi-square distribution for conditioning versus expectancy with female predominance versus female non-dominance (F = M and M > F), it yields significance (Fisher’s Exact test, *p* = 0.06, one-sided).

## Behavioral Versus Physiological Responses

Of specific note is that none of the four studies on visceral placebo analgesia ever produced sex differences at the behavioral (pain report) level, but one showed sex-dependent brain correlates of a placebo intervention despite equal subjective pain reports (Theysohn et al., 2014): Women exhibiting stronger responses in some brain regions (insular, prefrontal cortex) in anticipation of pain, but lower downregulation of activation in the same areas during the pain, in contrast to men; this may be indicative of the known higher pain sensitivity of females. An early PET study had demonstrated that females when exposed to placebo show significantly greater brain activation in the prefrontal cortex, as compared to the males (Paulson et al., 1998). Further imaging studies showed that the (blinded) application of i.v. glucose induced dopamine and increased glucose binding in the striatum in men but not in women (Haltia et al., 2008) and differentially affected blood pressure between sexes (Haltia et al., 2007), underlining a similar mechanisms at the CNS level than the Theysohn et al. study. Sex differences have also been shown to exists for the opioid system (Niesters et al., 2010), further supporting and explaining these differential effects on the background of approved involvement of the opioid (Sauro and Greenberg, 2005) and dopamine system (Scott et al., 2007, 2008) in placebo analgesia.

Other neuro-endocrine mediators have been nominated to the placebo response, among the first were NO (Stefano et al., 2001; Fricchione and Stefano, 2005), oxytocin (Enck and Klosterhalfen, 2009), the endocannabinoid system (Benedetti et al., 2011), and CCK (Benedetti et al., 2006). While for the first (NO), an empirical prove has never been presented, the involvement and OXT has been shown (Kessner et al., 2013; Colloca et al., 2015; Tracy et al., 2017), though not without contradictory data: While OXT worked in enhancing placebo analgesia, especially in women (Colloca et al., 2015; Tracy et al., 2017), it did not in dermal itch (Skvortsova et al., 2018). Its greater action in women supports the behavioral finding of smaller effects in women in pain challenges, as compared to men: mere verbal suggestion of beneficial effects of presumed analgesics (in fact, placebos) is not sufficient to induce analgesia in women, but requires additional trust, mediated by OXT.

For CCK the involvement in nocebo hyperalgesia has only shown in one study so far (Benedetti et al., 2006), and for the endocannabinoid system, supporting evidence has been shown by Pecina et al. (2014). Specifically for placebo and nocebo effects of hypobaric pressure (high altitude) sickness symptoms, the involvement of prostaglandins has been shown (Benedetti et al., 2014), but neither of these neuroendocrine mediator produced differential effects between the men and women.

## Sex Effects on Experimenter – Volunteers Interactions

In a sham-acupuncture trial with one male and one female therapist, the female acupuncturist induced greater trust than the male in having received true acupuncture (White et al., 2003). In the re-evaluation of a RCT in 120 IBS patients, the female physician produced greater symptom improvements in the drug and the placebo arm of the trial than her two male colleagues, and more female than male patients responded to placebo (Enck et al., 2005). Both studies can point toward the potential role of sex of doctors in the placebo responses, but cannot prove it.

In an experimental pain study by Kallai et al. (2004) significant interaction of the sex of male and female experimenter (*N* = 4 each) and sex of male and female volunteers (*N* = 80 each) on pain tolerance (cold pressor test) indicated that subjects tolerated pain longer when investigated by an experimenter of opposite sex. A significant main effect was found for sex of the experimenter: higher pain intensities and higher pain tolerance were found with female experimenters.

The first experiment in a placebo research setting (Flaten et al., 2006) noted higher placebo analgesia in males than in females following verbal manipulation of expectancies – in this experiment they used five female nurses as experimenters. To further explore sex differences on pain perception, they included experimenters of both sexes (*n* = 3 each) in another experiment (Aslaksen et al., 2007) and found significant interaction between both factors, in that female experimenters produced higher placebo analgesia in male volunteers than in females, while male experimenters did not produce similar responses, neither with male nor with female participants. This was not reflected in physiological data (heart rate), indicating – so the authors – the sex effect seen is probably due to psychosocial factors. In a third placebo analgesia experiment, this time with 8 experimenters (4 females), and with 64 volunteers (32 females), equally distributed in a balanced fashion, the dominant male response to female experimenters was not replicated. Instead significant sex (experimenter) × sex (volunteers) with a larger placebo analgesic response in males reporting to male experimenters, compared with male subjects reporting to female experimenters. With respect to pain reports (but not to placebo analgesia) the influence of experimenter sex persisted, however, male participants reported lower pain to female experimenters compared with the male experimenters in line with previous studies, as is a significant main effect of experimenter sex, with lower pain reports to female experimenters than to male experimenters.

Further evidence for a sex-by-sex interaction comes for two other placebo experiments, however, except Flaten et al. (2006), neither study has varied systematically the number and sex of the experimenters, and it may well be that the effects seen are therefore not sex- but personality-linked. Stumpf et al. (2016) noted no sex difference in the placebo response for itch, but a difference between the one male and female investigators, with respect to the exaggerated verbal suggestion and the respective control conditions, with the female experimenter producing higher flares size in the histamine condition. In a nausea study by Weimer et al. (2012) that provided verbal information of the anti-emetic effect of ginger (placebo), men who received placebo responded stronger to placebo information when provided by the male experimenter, and to ginger information when provided by the female experimenter; such effect was not seen in females. One explanation provided by the authors is that women’s behavior is stronger connected to their symptoms (and to information provided) than men’s behavior.

## Why Appear Pain and Nausea Prone to (Opposite) Sex Differences in Placebo/Nocebo Response?

Placebo and nocebo effects, as has been shown by many experimental investigations, can reliably be elicited in healthy volunteers, with many experimental paradigms, verbally induced or conditioned, but specifically with pain and nausea. At the same time, only pain and nausea have been shown to reliably be effected by sex, and two opposite conclusions can be drawn from the above discussed data:

(1) Despite higher sensitivity toward pain in females, placebo analgesia is easiest to elicit verbally in males.
(2) Conditioning is specifically effective to elicit nocebo effects, and works specifically well in females and with nausea.

For both conclusions, a rational concept is needed, despite the fact that they are based on only a few experiments from only a few placebo research groups, not necessarily interested in sex and gender differences *per se*.

For one, the above (Tables 3, 4) displayed distribution of research paradigms may be biased by an arbitrary or rational selection processes: Investigating placebo analgesia (instead of placebo responses in other areas of medicine) is determined – among others – by the simplicity of testing pain under laboratory conditions through a variety of techniques, that all (or many) also allow exportation into brain scanners and other advanced research technology. As we have elucidated before (Enck et al., 2018), our own decision to focus on nausea and a rotation paradigm was made before this was labeled placebo research (in 2004), as was our interest in sex differences, e.g., of nausea susceptibility (Stockhorst et al., 1998; Klosterhalfen et al., 2000).

Both pain and nausea were among the earliest clinical conditions that gain interest for their strong placebo responsiveness, as early reports from Beecher (1955) and Wolf (1959) indicate. At the same time, pain as well as nausea are among the most frequent symptoms reported in medicine, be it in clinical practice as subjective symptom in many somatic and functional diseases (Enck et al., 2016, 2017), or as adverse events or patient reported outcomes in RCT of drugs and other interventions, also in the placebo arms of trials (Rief et al., 2006). At the same time, both symptoms lack a biological correlate (biomarker) that can be used reliably to measure it, so that medicine is still relying on subjective assessment of its nature (threshold, tolerance, intensity) (Weimer et al., 2014; Saltychev et al., 2016).

Both symptoms are not *per se* diseases by their own, but rather indicative of an underlying process that requires medical attention and explanation, and only as a chronic condition (without such a process) become markers of a disease, as chronic pain or recurrent nausea and vomiting. Nausea has been called an maladaptation symptom, e.g., in the context of motion sickness (Lackner, 2014). For women, especially nausea has an additional health relevance not apparent for men: Nausea may be indicative of pregnancy at an early stage, and may serve as a biological warning signal in the interest of the safety of the unborn life, that has overcome from evolution.

The apparent difference between men and women with regards nausea on the one hand, and to verbally induced or conditioned responses on the other hand is best illustrated by the Klosterhalfen et al. (2009) experiment where we showed that women respond to conditioning of nausea symptoms much better than men, while men were more susceptible toward verbally induced symptom provocation. The obvious interpretation of these differences is that for women, learning mechanisms dominate – and previously learned content remains relevant -, while in men, an acutely provided information is of higher relevance than past experiences. This may also explain the higher susceptibility of men for verbally induced placebo analgesia, despite their lower overall pain sensitivity.

Three more experiments from our pre-placebo research tradition may further illustrate the importance of sex for nausea experience: In a study using a circular-vection drum to induce nausea (Klosterhalfen et al., 2008), we found that women responded stronger to the stimulus while sitting, while in men, the lying position was much more aversive. Significant differences between sexes were also found for habituation to repetitive rotation exposure: both endocrine and inflammatory markers habituated differently between men and women with multiple (five) rotations on the same day: increases in men and decreases in women in the first session versus increases in men and in women in the last session (Rohleder et al., 2006). With rotations repeated over (five) consecutive days (Meissner et al., 2009), males responded stronger on day 1 and reduced responses on days 2 and 3, while women responded stronger on day 3, as compared to days 1 and 2. For days 4 and 5, these trends reversed, again differentially between sexes.

All these data has led us to believe that both psychological and biological factors contribute to nausea reports in these experimental situations and interaction in rather complex ways, and presumably involving other factors that our experiments did not completely control for (Klosterhalfen et al., 2005, 2006).

## The Apparent Discrepancy Between RCT and Experiments Requires an Explanation

In 2012, Franconi et al. (2012) stated that the available data are too preliminary in order to reach to a definitive conclusion, but that a sex effect on placebo responses is conceivable. In 2013, Weimer et al. (2015) found that sex effects on placebo responses in RCT across medicine and its subspecialties are not visible and can therefore be ignored. A few years later the evidence has substantially strengthened for sex effects in experimental work on placebo and nocebo effects, as we show above, but still remains poor for clinical RCT data. This apparent discrepancy between RCT and experimental data also needs an explanation.

The best explanation that we can provide today is referring to the different nature of experiments on the one hand and RCT on the other. In a well-planned experiment, the separation of expectancy manipulation and learning/conditioning – as the two main underlying mechanisms of the placebo response – can be achieved, and the relative contribution of either can be explored. For instance, this allowed Colloca and Benedetti (2009) and others, to directly compare the relative potency of a novel learning mechanisms for placebo analgesia (by social observation) to the other two (expectation and conditioning).

In a randomized placebo-controlled trial, in contrast, the amount and degree of factors referring to patients’ learning (medical history, previous therapies and their success and/or failure, duration of knowing the treating doctor, etc.) and to expectancies delivered and associated with the treatment (informed consent and AE reports, symptom diaries, number and intensity of doctor-patient contacts etc.) is neither known nor balanced, and may vary from patient to patient as well, e.g., in relation to his/her social environment and the “placebo by proxy” influences (Grelotti and Kaptchuk, 2011). Under these circumstances it is conceivable that any existing differences in placebo responsiveness between the sexes are averaged out in RCT, and result in equally sized placebo effects in men and women, as we have seen.

## Author Contributions

PE and SK had the idea for the paper and wrote the manuscript. PE extracted the literature.

## Conflict of Interest Statement

The authors declare that the research was conducted in the absence of any commercial or financial relationships that could be construed as a potential conflict of interest.
